# Deracemisation and stereoinversion by a nanoconfined bidirectional enzyme cascade: dual control by electrochemistry and selective metal ion activation†

**DOI:** 10.1039/d2cc03638j

**Published:** 2022-09-30

**Authors:** Beichen Cheng, Rachel S. Heath, Nicholas J. Turner, Fraser A. Armstrong, Clare F. Megarity

**Affiliations:** Department of Chemistry, University of Oxford, South Parks Road Oxford OX1 3QR UK clare.megarity@manchester.ac.uk; School of Chemistry, University of Manchester, Manchester Institute of Biotechnology 131 Princess Street Manchester M1 7DN UK

## Abstract

The unique ability of the ‘electrochemical leaf’ (e-Leaf) to drive and control nanoconfined enzyme cascades bidirectionally, while directly monitoring their rate in real-time as electrical current, is exploited to achieve deracemisation and stereoinversion of secondary alcohols using a single electrode in one pot. Two alcohol dehydrogenase enzymes with opposing enantioselectivities, from *Thermoanaerobacter ethanolicus* (selective for *S*) and *Lactobacillus kefir* (selective for *R*) are driven bidirectionally *via* coupling to the fast and quasi-reversible interconversion of NADP^+^/NADPH catalysed by ferredoxin NADP^+^ reductase – all enzymes being co-entrapped in a nanoporous indium tin oxide electrode. Activity of the *Lactobacillus kefir* enzyme depends on the binding of a non-catalytic Mg^2+^, allowing it to be switched off after an oxidative half-cycle, by adding EDTA – the *S*-selective enzyme, with a tightly-bound Zn^2+^, remaining fully active. Racemate → *S* or *R* → *S* conversions are thus achieved in high yield with unprecedented ease.

## Abstract 2

Enzymes nanoconfined in a porous electrode are electrochemically driven for deracemisation and inversion with additional control by metal ion activation.

## Graphical abstract

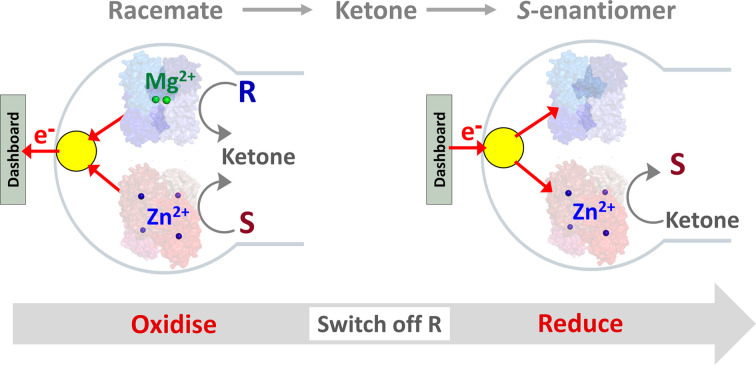

The ‘electrochemical leaf’ (e-Leaf) greatly extends protein film electrochemistry to include the ability to drive and control enzymes lacking an electron-transfer role. In the e-Leaf, ferredoxin NADP^+^ reductase (FNR) is bound tightly at high local concentration in the nanocavities of a highly porous indium tin oxide (ITO) layer formed on a conducting support. Under potential control, fast electron exchange between its active site flavin and the electrode results in the rapid bidirectional interconversion of NADP^+^/NADPH which is immediately coupled to a co-entrapped NADP(H)-dependent dehydrogenase.^1–3^ Extended enzyme cascades can also be loaded into the electrode pores where they are driven *via* this coupling which requires only one of the enzymes to be NADP(H)-dependent.^4^ The crowded environment mimics the nanoconfinement of metabolic enzyme cascades in biology. The unique combination – retention of intermediates and cofactors, the ability to energise and to control the direction and rate of the reaction, combined with real-time monitoring (which allows immediate observation of the response to interventions, such as addition^5^ or removal by, for example, replacement of the bulk solution^2^ or simply by chelation,^6^ of an effector) has already been exploited in several ways.^5,7,8^ Examples include the energisation and control of an extended five-enzyme cascade,^4^ monitoring of slow binding drugs to isocitrate dehydrogenase,^6^ and a two-step deracemiser.^9^ Most recently, a four-enzyme cascade, simultaneously driven by electrical and chemical energy, incorporated *in situ* ATP-recycling.^10^ The e-Leaf electrode is represented by the notation (E1 + E2…)@ITO/support, where E1 = FNR, E2 is a dehydrogenase and the support is graphite or titanium foil (the latter allowing double-sided electrophoretic deposition of ITO).

Most molecules in living organisms are chiral and therefore the binding of a drug is highly enantiomer-specific, exemplified by Thalidomide.^11–13^ For the pharmaceutical industry, effective deracemisation is therefore paramount. Our previous use of the e-Leaf for deracemisation^9^ sought to overcome compatibility problems encountered in common approaches.^14–22^ An alcohol dehydrogenase (ADH) pair with opposing enantioselectivities was used to resolve a racemic mixture in two steps.^9^ This work revealed an important consequence of rapid and reversible enzyme reactions occurring in the nanopore environment: local equilibrium is quickly attained, thereby favouring racemisation rather than deracemisation. A two-electrode system was thus needed to separate the oxidation and reduction stages.^9^

Here, we present a new strategy for deracemisation and stereoinversion, which exploits the different metal ion selectivities of two alcohol dehydrogenases loaded into the ITO electrode. Activity of the *R*-specific enzyme conveniently depends on the binding of Mg^2+^ ions, which allows it to be switched off at the half-way stage of an oxidation/reduction cycle, simply by adding EDTA; the other ADH in the pair (the active site of which contains a tightly-bound Zn^2+^) remaining unaffected (ESI,† Section S5). With this dual control, deracemisation or stereoinversion at high yield are thus achievable in the simplest way, a one-pot, one-electrode electrolyser.

Scheme 1 outlines the concept: FNR and an *S*-selective and *R*-selective ADH are nanoconfined in the porous electrode. When starting with a racemic mixture of alcohol, an oxidising potential is applied to drive conversion to the ketone, each enantiomer being processed by its respective ADH. The progress of the reaction is monitored until the current approaches zero and the charge passed equates to the amount of ketone produced. At this point, the *R*-selective ADH is de-activated by adding EDTA to chelate Mg^2+^, the electrode thus becoming strongly *S*-selective. The potential is then reversed to drive the reduction of the ketone back to *S*-alcohol, by the *S*-selective ADH, thus resolving the racemic mixture. An analogous approach is taken to undertake the stereoinversion, *R* → *S.*

**Scheme 1:**
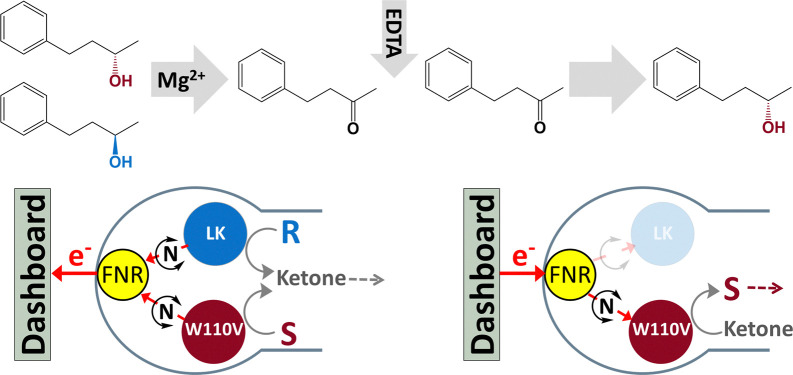
Deracemisation by dual control of ADH enzymes with opposing enantioselectivities, nanoconfined and driven in the porous electrode of the e-Leaf. The dashboard represents the potentiostat and computer for potential control and real-time monitoring. (For stereoinversion, the starting racemic mixture is replaced with *R*-alcohol.)

The important differences between the two ADHs are highlighted in Fig. 1. The *S*-selective variants which derive from *Thermoanaerobacter ethanolicus*, are homo-tetramers, each monomer containing a tightly bound catalytic Zn^2+^. The tryptophan at position 110 is exchanged for either alanine or valine, the latter having superior *S*-selectivity (they are referred to as W110A and W110V throughout).^23,24^ The *R*-selective enzyme (referred to as LK) which is also a homo-tetramer, derives from *Lactobacillus kefir*. This enzyme uses a catalytic triad instead of Zn^2+^ at the active site, but exhibits a strong dependency on non-catalytic Mg^2+^ for activity^25,26^ Hypotheses on the role of Mg^2+^ are outlined in Section S2 ESI,†.

**Fig. 1:**
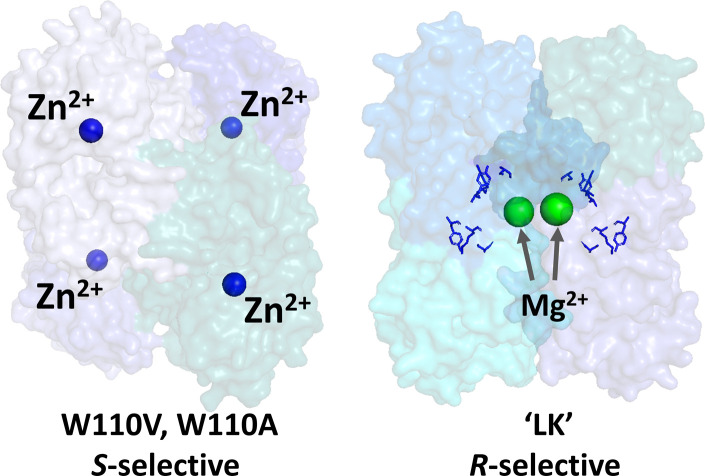
Alcohol dehydrogenases with opposing enantioselectivities. Left: *S*-selective ADH from *Thermoanaerobacter ethanolicus*, catalytic Zn^2+^ shown as blue spheres (PDB 7JNS). Right: *R*-selective ADH from *Lactobacillus brevis*, structural Mg^2+^ ions shown as green spheres and the catalytic triad residues, Ser-Tyr-Lys, shown in blue; this enzyme lacks Zn^2+^ (PDB 6H07).

Table 1 lists the kinetic data and enantiomeric preferences for each enzyme used in this study. The LK enzyme has no measurable activity with *S*-alcohols. Its high *R*-selectivity arises because during a catalytic cycle, the *pro-R* hydride from the nicotinamide is transferred to the si face of the carbonyl to give *R*-alcohols. Rotation of a bound ketone around its carbonyl bond such that its re face would be positioned to accept the hydride, would result in extreme steric hinderance.^25,27^ The relatively high *S*-selectivity of W110A is increased further by incorporating the bulkier residue, valine. This mutation renders the enzyme inactive for *R*- but also less active for *S*-alcohols.

**Table 1:** Kinetic Data for the ADH variants from *T. ethanolicus* (W110A and W110V) and the *R*-selective ADH from *L. kefir* (LK)

		W110A	W110V	LK
*S*	*k*_cat_/s^−1^	29.8^9^	6.2^9^	**—**
	*K*_m_/mM	0.50^9^	0.13^9^	**—**
*R*	*k*_cat_/s^−1^	0.68^9^	—	29.0^9^
	*K*_m_/mM	0.57^9^	—	1.58^9^
Ketone	*k*_cat_/s^−1^	48.4^9^	20.1^9^	32.8^9^
	*K*_m_/mM	0.29^9^	0.12^9^	0.13^9^
	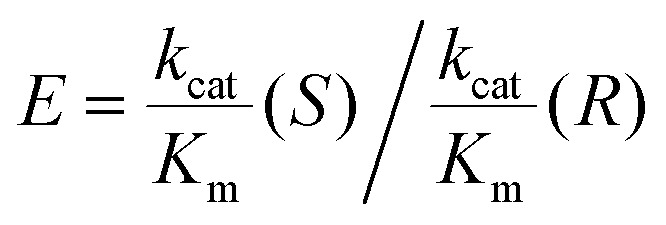	17.4^28^	134.5^28^	—

Fig. 2 shows the stereoinversion of (*R*)-4-phenyl-2-butanol contained initially at a concentration of 4 mM in 5 mL of aqueous solution (20 μmoles). The (FNR + LK + W110V)@ITO/Ti foil electrode (7 cm^2^) was prepared by preloading with FNR (drop-casting a concentrated solution) after which LK and W110V were loaded from a dilute solution containing 1.28 nmoles of LK and 0.66 nmoles of W110V. The electrode was rinsed thoroughly before use to remove unbound enzymes. The starting solution (pH 9.0, chosen to favour oxidation during the first stage^9^) also contained 5 mM Mg^2+^. Holding at a potential of +0.21 V *vs.* SHE, the oxidation half-cycle was initiated by introducing NADP^+^ to a final concentration of 20 μM, which resulted in a rapid increase in oxidation current attributable to the conversion of (*R*)-4-phenyl-2-butanol to 4-phenyl-2-butanone by LK. After approximately 24 h, when the current had decreased to a low level, the potential was set to open circuit and a sample was analysed by chiral GC. The result showed (Table S3 ESI,†) that 85.7% of the alcohol had been converted to ketone, the remainder being unreacted *R*- and a small amount of *S*-alcohol. The charge passed during the oxidation phase equated to the conversion of 17.0 μmoles (equivalent to 85% of the starting alcohol, in good agreement with the GC analysis which showed a conversion of 17.14 μmoles). The total number of turnovers undergone by each (averaged) NADP^+^ at this stage was 170.

**Fig. 2:**
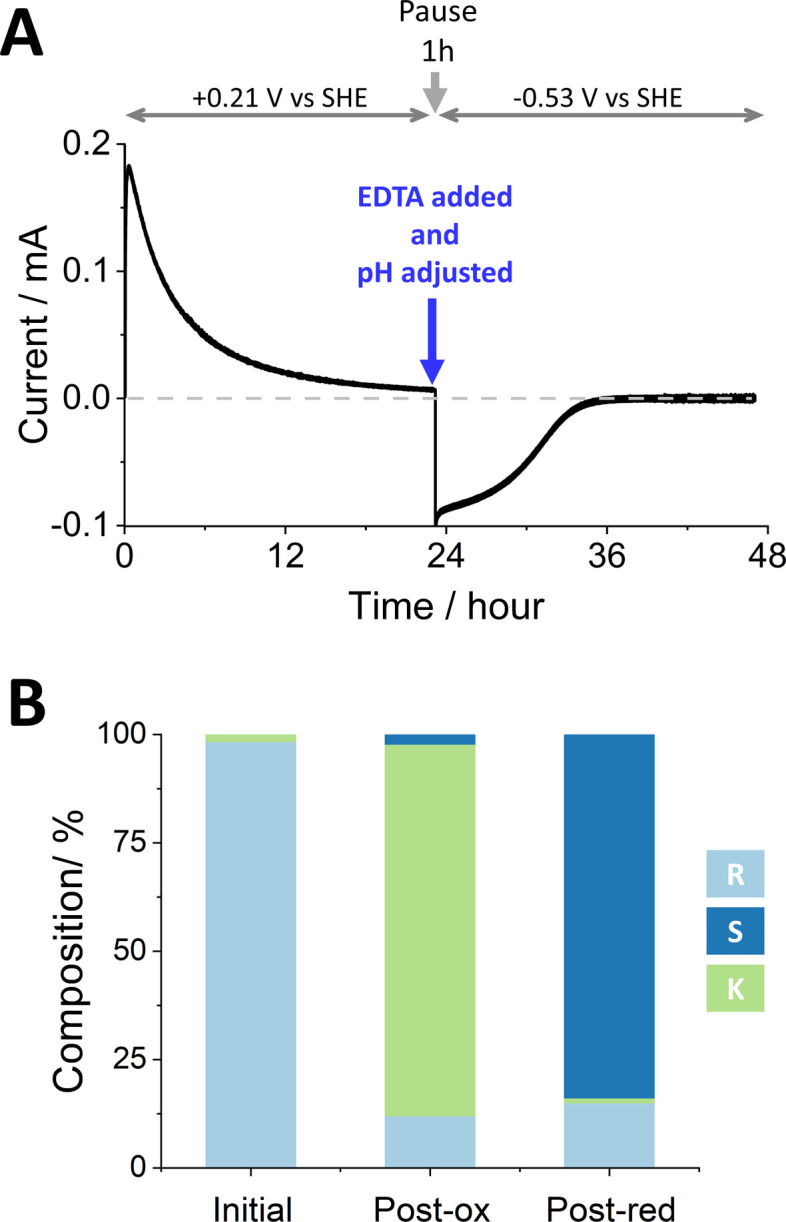
The stereoinversion of (*R*)-4-phenyl-2-butanol by W110V and LK nanoconfined in the e-Leaf. (A) As monitored by chronoamperometry following the addition of NADP^+^ at *t* = 0. At the end of the oxidation phase, a 1 mL sample was removed for chiral GC analysis and by a single addition, the pH was adjusted to 8 and EDTA was introduced to chelate Mg^2+^ thus inactivating LK (the reaction was paused for 1 h to ensure complete chelation). The potential was then switched to drive reduction, and measurement resumed. (B) Bulk solution compositions analysed by chiral GC. Conditions: initial cell volume 5 mL; solution stirred by magnetic flea; substrate [(*R*)-4-phenyl-2-butanol] = 4 mM, [NADP^+^] = 20 μM, [Mg^2+^] = 5 mM; buffer: 50 mM TAPS pH 9.0 at the start, adjusted to 8 after phase one; temperature: 25 °C; potential (*vs.* SHE) held at +0.21 V for oxidation and −0.53 V for reduction. An anaerobic glovebox was used to avoid contribution to the current from reduction of O_2_. Enzyme loading: FNR loaded first by drop-casting, LK and W110V loaded after FNR, by stirring in a 2.5 mL solution containing 1.28 nmols of LK and 0.66 nmols of W110V in 50 mM TAPS buffer pH 9; electrode (ITO@Ti): 7 cm^2^.

To prepare for the reduction phase, EDTA was added (to give a final concentration of 25 mM) and the pH was adjusted to 8.0 (to favour reduction^9^). After a pause lasting 1 h to ensure complete chelation of Mg^2+^ and inactivation of LK (the EDTA causes release of Mg^2+^ from LK but not release of the Zn^2+^ from W110V), an electrode potential of −0.53 V *vs.* SHE was applied, resulting in a rapid increase in reduction current which gradually decreased to zero after approximately 12 h. The total charge passed during the reduction phase equated to the conversion of 11.4 μmoles of ketone. Analysis of the main cell solution by chiral GC showed 2.78 mM (*S*)-4-phenyl-2-butanol, and 0.50 mM (*R*)-4-phenyl-2-butanol, corresponding to 11.1 and 2.0 μmoles, respectively. The solution in the side arm was also analysed by chiral GC to quantify material that had ‘crossed over’ through the porous frit. The combined data are shown in Table S3 (ESI†), which includes % conversion at each step along with ee% values. The total number of turnovers undergone by each (averaged) NADP^+^ throughout the two stages was 313.

Fig. 3 shows an experiment to undertake the deracemisation of an 8 mM solution of (±)-4-phenyl-2-butanol. The (FNR + LK + W110V)@ITO/Ti foil electrode (14 cm^2^ total area) was prepared as for the stereoinversion experiment shown in Fig. 2, and the solution, at pH 9.0, contained 5 mM Mg^2+^. Upon initiation of the oxidation phase by addition of NADP^+^, there was a rapid increase in current which gradually approached zero after approximately 40 h. The longer time taken compared to stereoinversion is explained by the inherently slow rate of W110V, required for deracemisation but inconsequential to the stereoinversion experiment where only LK is required for oxidation of the starting *R*-alcohol substrate.

**Fig. 3:**
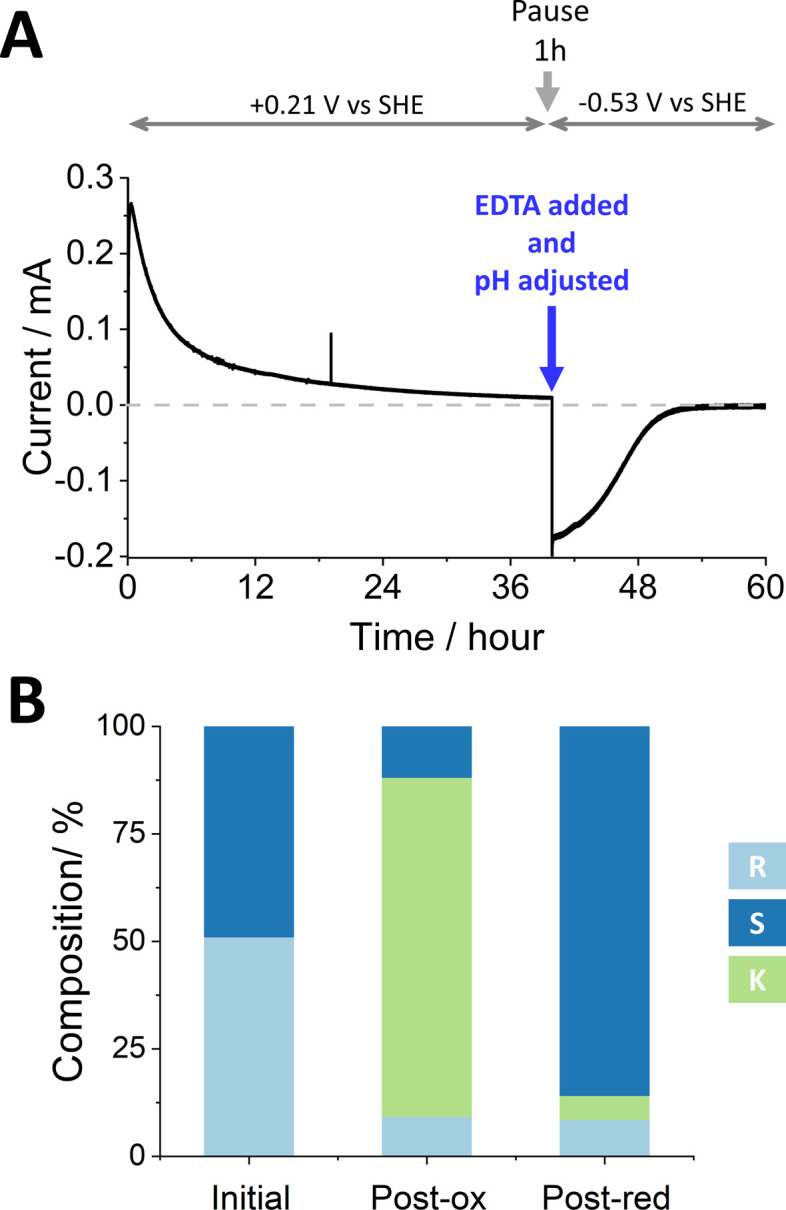
Deracemisation of a racemic mixture of (±)-4-phenyl-2-butanol by nanoconfined W110V and LK. (A) The reaction course monitored by chronoamperometry. Experimental steps carried out as for Fig. 1. (B) Bulk solution composition analysed by chiral GC. Conditions: as for Fig. 2 apart from starting substrate: [(±)-4-phenyl-2-butanol] = 8 mM and amount of ADH enzymes: 2.58 nmols of LK and 1.31 nmols of W110V; electrode (ITO@Ti): 14 cm^2^.

The charge passed during the oxidation phase equated to the conversion of 33.4 μmoles of reactant (83.5% of the quantity of (±)-4-phenyl-2-butanol). For comparison, GC analysis showed 4-phenyl-2-butanone at a concentration of 6.31 mM (31.6 μmoles; 78.9% conversion). The 5% discrepancy is explained in part by the crossover of ketone into the side arm which was not analysed by chiral GC at the midway point. Despite the current approaching zero, oxidation was incomplete since both *R*- and *S*-alcohols were still present at 0.73 mM and 0.95 mM respectively (Fig. 3B). We return to this result later.

The reduction phase was carried out as for Fig. 2, after injecting EDTA to give a final concentration of 25 mM, adjusting the pH to 8.0, and applying a potential of −0.53 V *vs.* SHE after a 1 h pause. The current dropped to zero after approximately 10 h. The total charge passed during this phase equated to a conversion of 21.1 μmoles. Analysis of the main cell solution by chiral GC showed 5.06 mM (*S*)-4-phenyl-2-butanol, and 0.50 mM (*R*)-4-phenyl-2-butanol, corresponding to 20.2 and 2.0 μmoles, respectively. The side-arm solution was also analysed. The combined data for this experiment are shown in Table S4 (ESI†), which includes conversions to % conversion at each step along with ee% values. The total number of turnovers undergone by each (averaged) NADP^+^ throughout the two stages was 598.

Equivalent experiments using W110A are shown in Section S3, ESI.† The stereoinversion experiment using this variant gave an inferior outcome overall, with more ketone remaining (11.3% *vs.* 1.1%) and less *S*-alcohol (75.5% *vs.* 83.9%). As shown in Fig. 2, use of the W110V variant, optimising for the reduction by pH adjustment midway, and ensuring complete inactivation of LK by prolonged chelation time, resulted in a better outcome. For deracemisation, any distinction between W110A and W110V was less clear. Despite having slightly more *S*-alcohol (86% *vs.* 84%) and less ketone (5.6% *vs.* 9.5%) in the final solution, use of W110V resulted in a similar enantiomeric excess to that achieved with W110A (ee%: 82 *vs.* 85.7). In all experiments, the *R*-enantiomer persisted to some extent. As proposed previously, an important factor may be the crowded nanoporous environment that traps reactants and products for sufficient time to accelerate the approach to final equilibrium, which always favours a racemic mixture.^9^

In this proof-of-concept, we have exploited, (i) the ability to drive ADH enzymes with opposing enantioselectivities, bidirectionally, (ii) the ability to inactivate, selectively, the *R*-specific enzyme, (iii) the ability to monitor the reaction in real-time to guide intervention and (iv) the advantages of nanoconfinement for enzyme cascades. A new approach for one-pot deracemisation and stereoinversion using a single electrode is thereby demonstrated. Although high enantiomeric excesses have already been achieved, 82–85.7%, the many factors at play – inherent enzyme rates, *K*_m_ values and enantiomeric selectivity – mean there is considerable scope for improvement and bespoke optimisation.

The proof-of-concept can be taken forward in various ways, for example, an FNR variant specific for NAD(H) rather than NADP(H), will expand the scope. The platform is well suited to selective hydrogenation and deuteration. The emerging field of *de novo* enzyme production, through computational design and directed evolution,^29^ will expand the toolkit further; for example, the use of variants engineered to have built-in on-off “switches” to facilitate differential control.

C. F. M conceived the project, B. C. carried out all experiments, R. H. and N. J. T. provided *T. ethanolicus* variants, R. H. carried out chiral analysis, F. A. A., B. C., C. F. M. interpreted the data and wrote the manuscript.

This research was supported by a grant from the Biological and Biotechnological Research Council (Follow-on Fund, Grant BB/P023797/1), the EPA Cephalosporin Fund (CF 342) and by an ERC Advanced Grant (Grant number 742987).

## Conflicts of interest

There are no conflicts to declare.

## Supplementary Material

CC-058-D2CC03638J-s001

CC-058-D2CC03638J-s002

CC-058-D2CC03638J-s003

CC-058-D2CC03638J-s004

CC-058-D2CC03638J-s005

CC-058-D2CC03638J-s006

CC-058-D2CC03638J-s007

CC-058-D2CC03638J-s008
