# Supplementary figures and images for: Deracemisation and stereoinversion by a nanoconfined bidirectional enzyme cascade: dual control by electrochemistry and selective metal ion activation
Source: Chem Commun (Camb). 2022 Sep 30;58(83):11713–6. doi: 10.1039/d2cc03638j (PMC9578339; doi:10.1039/d2cc03638j)

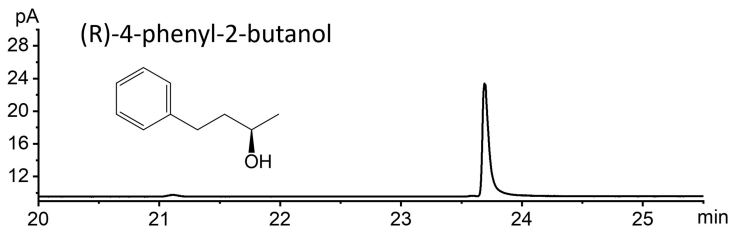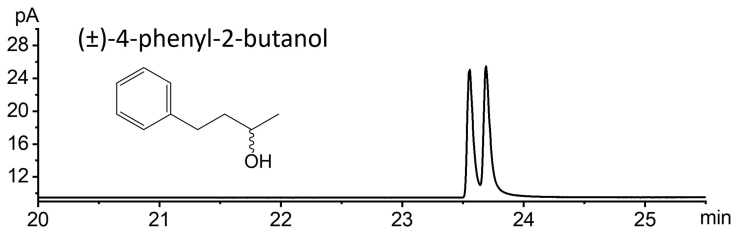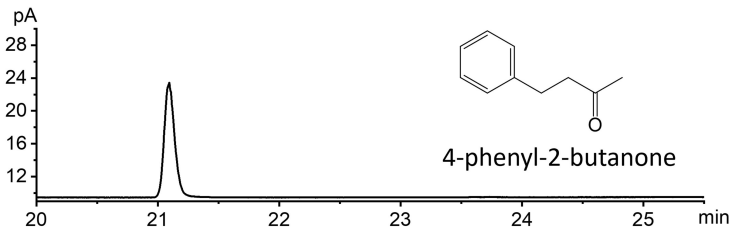

Supplement: CC-058-D2CC03638J-s002 [file CC-058-D2CC03638J-s002.pdf]

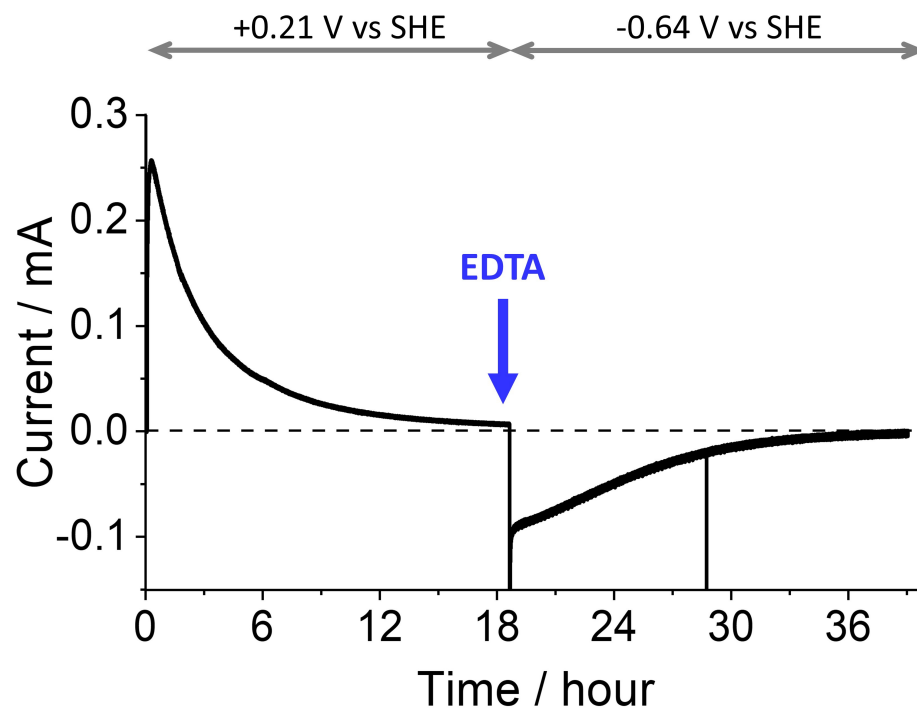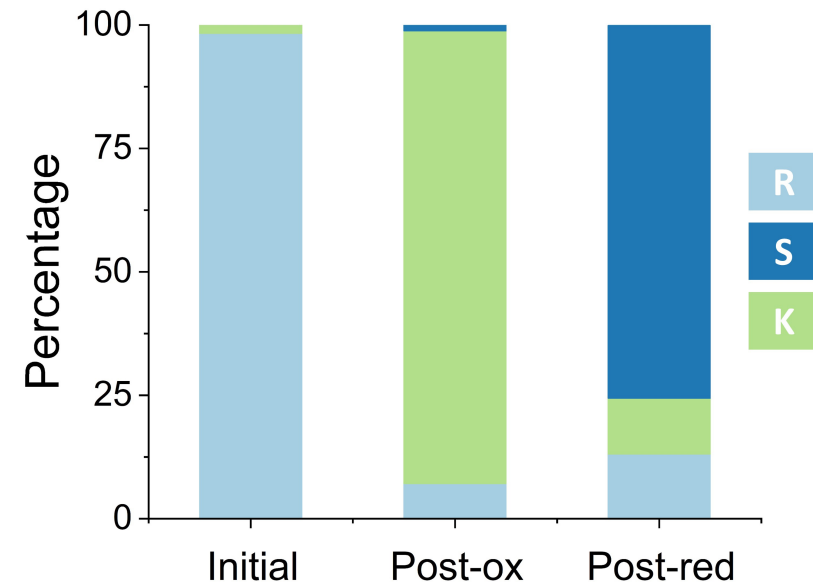

Supplement: CC-058-D2CC03638J-s003 [file CC-058-D2CC03638J-s003.pdf]

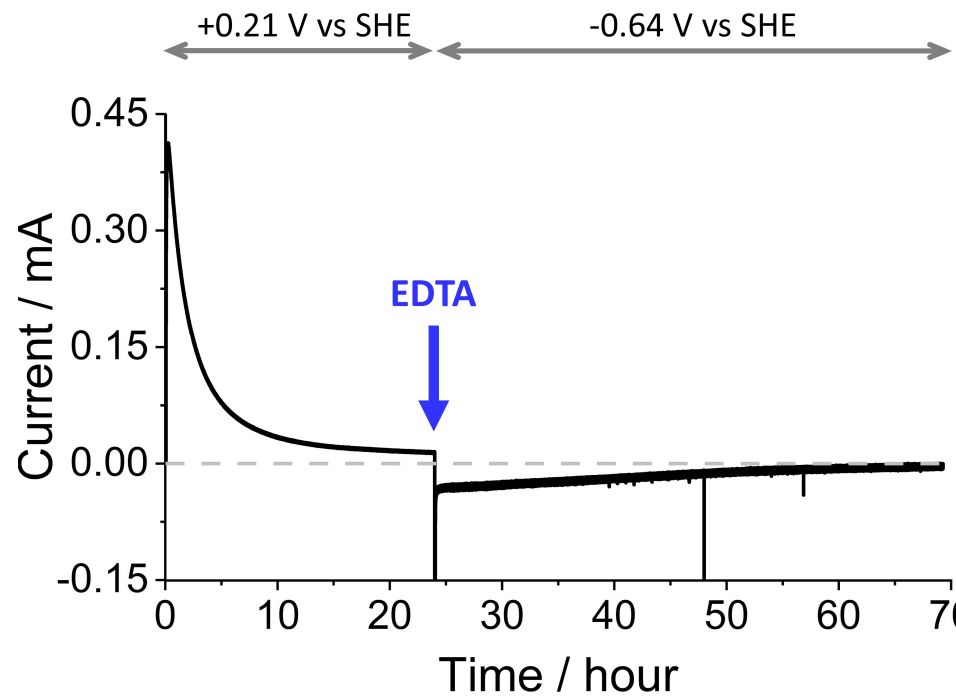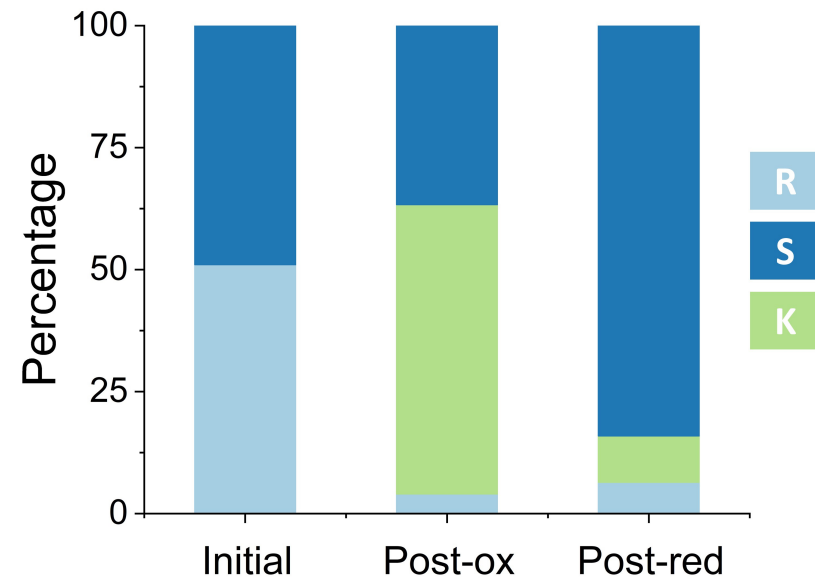

Supplement: CC-058-D2CC03638J-s004 [file CC-058-D2CC03638J-s004.pdf]

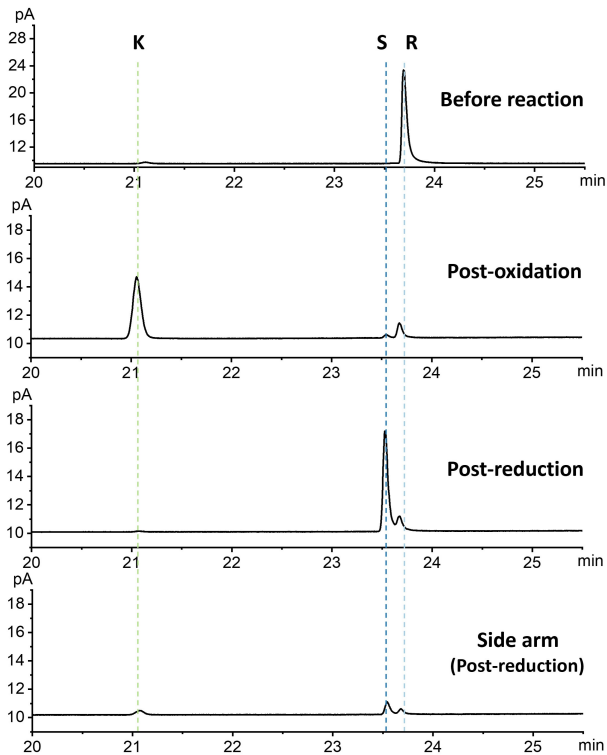

Supplement: CC-058-D2CC03638J-s005 [file CC-058-D2CC03638J-s005.pdf]

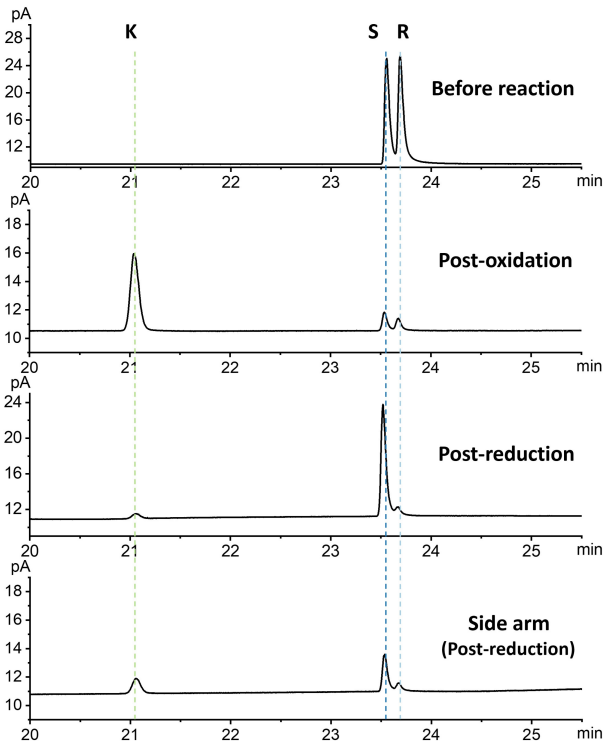

Supplement: CC-058-D2CC03638J-s006 [file CC-058-D2CC03638J-s006.pdf]

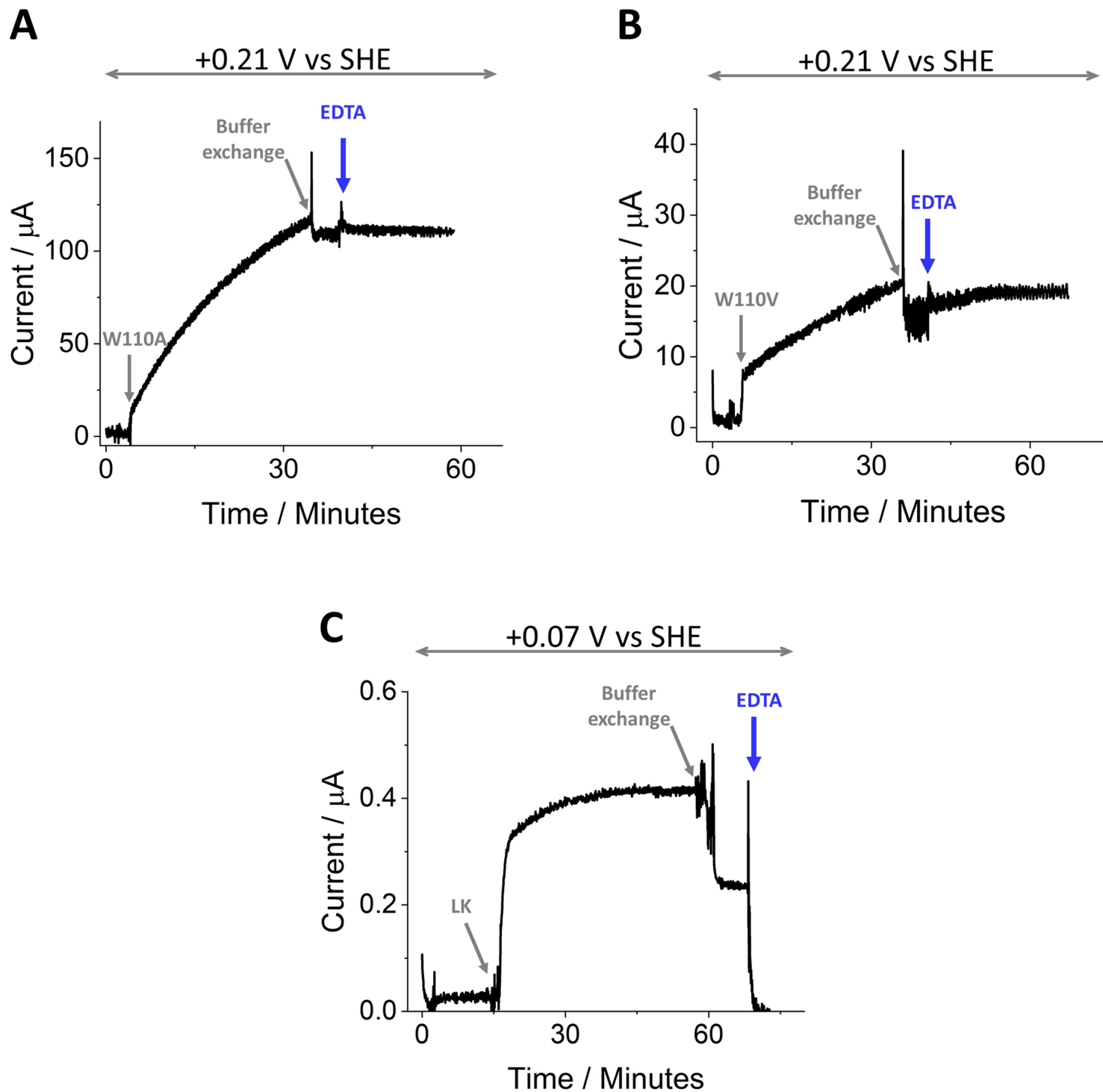

Supplement: CC-058-D2CC03638J-s007 [file CC-058-D2CC03638J-s007.pdf]
