# Supplementary material for: Deracemisation and stereoinversion by a nanoconfined bidirectional enzyme cascade: dual control by electrochemistry and selective metal ion activation
Source: Chem Commun (Camb). 2022 Sep 30;58(83):11713–6. doi: 10.1039/d2cc03638j (PMC9578339; doi:10.1039/d2cc03638j)
Supplement: CC-058-D2CC03638J-s008 [file CC-058-D2CC03638J-s008.pdf]

Electronic Supplementary Material (ESI) for Chemical Communications.
This journal is © The Royal Society of Chemistry 2022

Electronic Supplementary Information

Deracemisation and Stereoinversion by a Nanoconfined Bidirectional Enzyme Cascade: Dual Control by Electrochemistry and Selective Metal Ion Activation

Beichen Cheng[a], Rachel S. Heath[b], Nicholas J. Turner[b], Fraser A. Armstrong*[a] and Clare F. Megarity*[a†]

Contents

Section 1: Methods 2
- Electrochemical Methods 2
- Electrode Construction 2
- Enzyme Loading 2
- Chronoamperometry Procedure 2
- GC Analysis 4
- Enzyme Purification 5
  - Ferredoxin $NADP^+$-Reductase (FNR) 5
  - ADH Variants from *Thermoanerobacter ethanolicus* (W110A and W110V) 5
  - ADH from Lactobacillus Kefir 6

Section 2: The Role of Magnesium Ions in the *Lactobacillus kefir* Enzyme 8

Section 3: Results of Preliminary Experiments Using W110A 9
- The stereoinversion of R-alcohol to S-alcohol 9
- The Deracemisation of Racemic Alcohol to S-alcohol 11

Section 4: Chiral GC Analysis for Figure 2 and Figure 3 in the Main Manuscript 13
- Enantiomer analysis for the stereoinversion experiment shown in Figure 2 13
- Enantiomer analysis for the deracemisation experiment shown in Figure 3 14

Section 5: Control experiments to establish that the Zinc-Dependent variants, W110A and W110V, remain active in the presence of 25 mM EDTA. 16

References 18

Section 1: Methods

Electrochemical Methods

To avoid a contribution to the catalytic current by the reduction of O_2, all electrochemical experiments were performed under anaerobic conditions in a glovebox (Belle Technologies) containing a N_2 atmosphere (O_2 < 2 ppm). A 3-electrode configuration in a 3-compartment glass electrochemical cell was used for all experiments: the platinum counter electrode was housed in an auxiliary side arm separated from the working reaction solution by a glass frit to minimise the crossover of products as they could be oxidised or reduced at the Pt; the reference electrode (AgAgCl, 3M KCl) was also contained in a separate compartment. The potentials used in the chronoamperometry experiments are quoted versus the standard hydrogen electrode (SHE) and were converted using the equation $E_{SHE} = E_{Ag/AgCl} + 0.21$ V.[1]

Electrode Construction

Electrodes consisted of titanium foil supports onto which ITO particles (Sigma-Aldrich < 50 nm) were electrophoretically deposited. The ITO nanopowder suspended in acetone with Iodine ((0.02g ITO nanoparticles + 0.01g Iodine in 20 mL acetone) and sonicated for at least 45 minutes The Ti foil to be modified was held in parallel to the counter electrode in the ITO suspension at a close distance apart (approximately 1 cm). A 10 V potential was applied for 7 minutes deposition on each side of the Ti foil. The ITO-coated electrode is allowed to dry, rinsed thoroughly in ultrapure water (Millipore, 18 MΩ cm) and allowed to dry before use.

Enzyme Loading

For all experiments, the enzymes were loaded into the pores of the ITO electrode as follows:

FNR was preloaded on the electrode by drop−casting: a small volume of a concentrated enzyme solution (typically 1.64 nmol FNR per cm^2 of electrode surface geometric area) was dropped directly onto the surface of the ITO surface and incubated for approximately 20-30 minutes taking care to prevent drying. The electrode was then thoroughly rinsed with buffer (50 mM TAPS pH 9) to remove any unbound enzyme. This FNR-loaded electrode was then placed in a dilute solution containing a specified ratio of the alcohol dehydrogenases and stirred in a cold room (4 °C) for several hours (typically between 5 h and 18 h, depending on the experiment), to allow the enzymes to adsorb into the pores alongside FNR. Immediately before use, the electrode was rinsed to remove any unbound enzyme.

Chronoamperometry Procedure

All electrochemical experiments took the form of chronoamperometry where the potential is held constant, and the rate is monitored directly and in real-time as electrical current. The overall approach (as shown in scheme I), involved firstly, an oxidation stage (potential held at +0.21 V vs SHE which is sufficiently positive of the alcohol/ketone reduction potential[2]) during which the ADH enzymes nanoconfined in the pores, are

driven to oxidise their corresponding alcohol enantiomers to ketone. The process is monitored, and the endpoint can be estimated as the current approaches zero. At this point, the potential is flipped to a reducing value (-0.53 V vs SHE, which is sufficiently negative of the alcohol/ketone reduction potential[2]; note for the experiments shown in S2 and S3, the reducing potential chosen was -0.64 V vs SHE) and the LK enzyme is inactivated by the addition of ethylenediaminetetraacetic acid disodium salt dihydrate (EDTA, CAS number: 6381-92-6) to chelate the magnesium which this enzyme requires for activity. The reductive part of the reaction thus proceeds with only the S-selective ADH, remaining active to reduce the ketone. For each experiment, the ratio of enzymes was chosen based on the aim, for example to favour (R)-selectivity in the oxidation stage whilst still ensuring sufficient activity by the W110A variant for the reduction stage. The whole process typically lasted for at least 30 hours, and the enzymes have proved to remain adsorbed and active throughout the process. As described in the following results section, further alterations for improved performance were made and include pH adjustment mid-process (previously established for the optimisation of conditions for consecutive oxidation and reduction reactions[2]) at the same time as the potential switch, and optimisation by increasing the electrode surface area and also the time allowed for deactivation by EDTA. The final cell solution (and samples of solution removed during the experiment) were analysed by chiral gas chromatography as described[2] with enantiomeric excess calculated using the equation: ee%(S) = $\frac{[S]-[R]}{[S]+[R]} \times 100$

GC Analysis

Samples taken for GC analysis were first extracted into methyl tert-butyl ether (MTBE) and analysed as described.[3] In brief, conversion and enantiomeric excess were determined by GC by GC-FID using a GC 6850 instrument fitted with an autosampler (Agilent) and a chiral BetaDex™ 325 column (30 m x 0.25 mm x 0.25 µm) (Supelco).

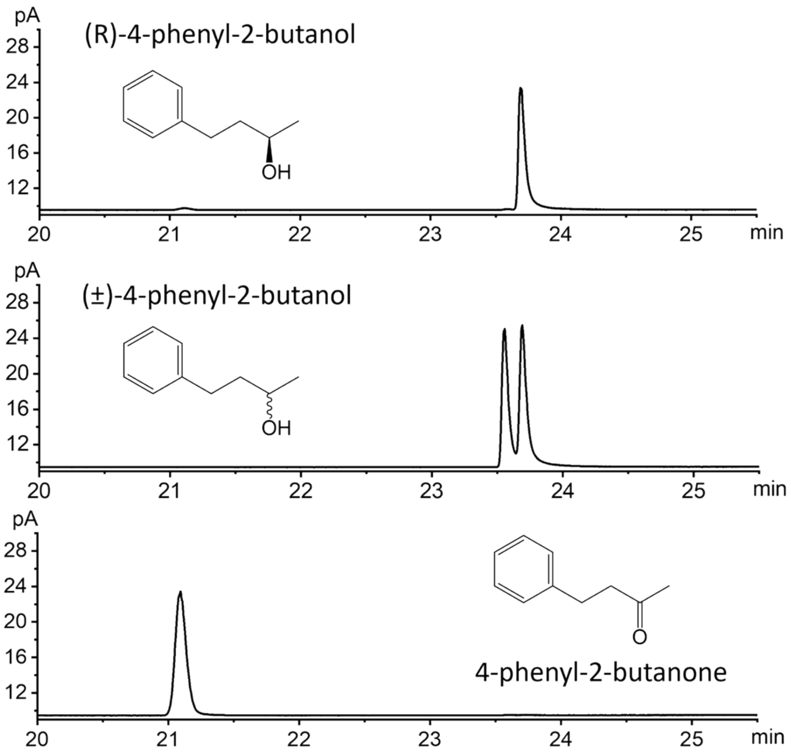

Figure S1. GC analysis results of standard solutions of (R)-4-phenyl-2-butanol, (rac)-4-phenyl-2-butanol and 4- phenyl-2-butanone (8 mM in 50 mM TAPS buffer, pH 9.0). GC protocol: inlet temperature 200 °C, detector temperature 250 °C, oven 110 °C/hold 20 mins; 10 °C/min to 200 °C. Rt (4-phenyl-2-butanone) = 20.8 minutes. Retention time: Rt((R)-4-phenyl-2-butanol) = 23.7 minutes, Rt ((S)-4-phenyl-2-butanol) = 23.6 minutes, Rt(4-phenyl-2-butanone) = 21.1 minutes. The concentration of the ketone and enantiomeric alcohols determined by GC-FID are shown in section 4.

Enzyme Purification

Ferredoxin NADP$^+$-Reductase (FNR)

The expression and purification of FNR was carried out as previously described[4] (outlined below).

Expression: A vector (aLICator pLATE 51) containing the gene encoding N-terminal Histagged FNR from *Chlamydomonas reinhardtii* was used to transform *Escherichia coli* cells (BL21 (DE3)) which were then plated on Lysongeny broth (LB) agar containing ampicillin at 0.1 mgmL^{-1}. Positive transformants were selected by resistance to ampicillin. A single colony was used to inoculate 100 mL LB media containing ampicillin at 0.1 mg mL^{-1} and grown shaking (200 rpm), overnight at 37°C. This was used to inoculate 500 mL of LB containing ampicillin at 0.1 mgmL^{-1} and grown at 37°C, 200 rpm for approximately 3 hours at which point they were induced by addition of isopropyl β-D-1-thiogalactopyranoside (IPTG) to a final concentration of 1 mM and grown for a further 3 – 4 hours. The cells were then harvested by centrifugation and the pellets resuspended in cold cell resuspension buffer (50 mM HEPES; 150 mM NaCl; 10% V/V Glycerol pH 7.4) and stored at –80°C until purification.

Purification: The cells were thawed and then disrupted using a French press at 20 psi; cell debris was removed by centrifugation at 45000 rpm for 1 hour (Beckman Ultracentrifuge). The supernatant was loaded onto a Ni^{2+} HisTrap affinity column (GE Healthcare Life Sciences) using an Äkta purification system with dual wavelength absorbance detector. The column was washed with 50 column volumes of buffer (50 mM HEPES, 500 mM NaCl, 1 mM dithiothreitol (DTT), pH 7.4. A linear concentration gradient of imidazole reaching a maximum of 250 mM in ~30 minutes at a rate of 1.5 mLmin^{-1}, was used to elute FNR in 1 mL fractions; those containing FNR were selected based on the absorbance at 280 nm and 460 nm.

The fractions were pooled and concentrated using centrifugal filters (Amicon® Ultra–4 Merck). The concentrated protein was passed through a desalting column (PD–10 Ge Healthcare) to remove imidazole, portioned into single use aliquots and flash frozen in liquid nitrogen before storing at –80°C.

ADH Variants from *Thermoanerobacter ethanolicus* (W110A and W110V)

Expression and purification of each variant were carried out as previously described[2] (outlined below)

Expression: A pET28 vector containing the gene for the alcohol dehydrogenase with mutation W110A (TesADH W110A) was transformed into *E. coli* BL21 (DE3) cells (New England Biolabs) following the manufacturer's protocol. Colonies were grown on LB agar containing 30 µgmL^{-1} kanamycin, overnight at 37°C. A single colony was used to inoculate 5 mL LB media containing kanamycin at 30 µgmL^{-1} and grown overnight at 37°C, 250 rpm. This overnight culture was then used to inoculate 600 mL of TB autoinduction media

(Formedium) containing kanamycin at 30 μgmL^{-1} in a 2 L baffled flask and incubated for 16 - 20 hours at 37°C and 250 rpm.

The cells were then harvested by centrifugation (4000 rpm, 30 mins, 4°C) and the cell pellets were resuspended in cold buffer (100 mM potassium phosphate, pH 7.7, 300 mM NaCl, 20 mM imidazole, 1 mM dithiothreitol (DTT)) and stored at –80°C until purification.

Purification: After thawing, the cells were disrupted three times using a French press at 20 psi and the debris removed by ultracentrifugation (Beckman Ultracentrifuge) for 1 hour at 45000 rpm and 4°C. The supernatant was loaded onto a 5 mL HisTrap FF column (GE Healthcare Life Sciences) using an Äkta purification system; the column was previously equilibrated with 10 column volumes of buffer (100 mM potassium phosphate, pH 7.7, 300 mM NaCl, 20 mM imidazole, 1 mM dithiothreitol (DTT) (buffer A). After enzyme loading, the column was washed with 10 column volumes of the buffer A. The enzyme was eluted using an imidazole gradient (0-100% buffer B (100 mM potassium phosphate, pH 7.7, 300 mM NaCl, 300 mM imidazole, 1 mM DTT) over 10 column volumes). Fractions containing the ADH enzyme were selected based on absorbance at 280 nm and were pooled and dialysed overnight against 1 litre of dialysis buffer (Tris (50 mM pH 8.0) supplemented with 1 mM DTT) at 4°C. The desalted enzyme was then concentrated using a 10 kDa molecular weight cut-off spin column (GE Healthcare), aliquoted and flash frozen in liquid nitrogen before storage at –80°C

ADH from Lactobacillus Kefir

The expression and purification of LK ADH was carried out as previously described[2] (outlined below).

Expression: The gene encoding ADH from *Lactobacillus kefir* was inserted into a vector, which also encoded an N-terminal hexa- HIStag and the genes to confer ampicillin resistance, using ligation independent cloning (LIC) (aLICator, Thermo Scientific, N-terminal His-tag/EK, #K1251); the sequence was confirmed by Source Bioscience. The plasmid was transformed into *E. coli* BL21 (DE3) cells (New England Biolabs) and grown overnight at 37°C on LB agar plates supplemented with ampicillin to a final concentration of 0.1mgmL^{-1}. A flask containing 100 mL LB (25 gL^{-1}) supplemented with ampicillin (0.1 mgmL^{-1}), was inoculated with a single positive colony and grown overnight at 37 °C and 250 rpm. This overnight culture was used to inoculate six 500 mL portions of LB (25 gL^{-1}) supplemented with ampicillin (0.1mgmL^{-1}) (approximately 15 mL of overnight culture was added to each 500 mL). The cells were grown at 37°C and 200 rpm for 2-2.5 hours until approximately mid-log phase upon which IPTG was added to a final concentration of 1.3 mM. The temperature was reduced to 30°C and overexpression of the protein was allowed to continue for approximately 20 hours (at 250 rpm). The cell cultures were then centrifuged at 6000 rpm for 30 minutes at

4 °C and the cell pellets resuspended in cold buffer (50 mM Tris, 500 mM NaCl, 1 mM $MgCl_2$, 10 % glycerol, pH 8.0).

Purification: The cells were disrupted using a French press (three times at 20 psi). Debris was removed by ultracentrifugation for 1 hour at 45000 rpm and 4°C and the supernatant collected and loaded onto a preequilibrated (with buffer A: 50 mM Tris, 500 mM NaCl, 1 mM DTT, 1 mM $MgCl_2$, pH 8.0) 5 mL His-Select Ni^{2+} column (GE Healthcare Life Sciences) using an ÄKTA protein purification system; the column was washed with 10 column volumes of buffer A. The enzyme was eluted in the same buffer by a linear gradient of imidazole to reach 250 mM (buffer B: 50 mM Tris, 500 mM NaCl, 1 mM DTT, 1 mM $MgCl_2$, 250 mM imidazole, pH 8.0) over 10 column volumes. Fractions containing LK ADH were selected based on absorbance at 280 nm, were pooled and then dialysed overnight against 1-2 litres of Tris buffer (50 mM pH 8.0) supplemented with 1 mM DTT. The enzyme was then concentrated using a 10 kDa molecular weight cut-off spin column (GE Healthcare), aliquoted and flash frozen in liquid nitrogen before storage at –80°C

Section 2: The Role of Magnesium Ions in the *Lactobacillus kefir* Enzyme

The R-selective enzyme is a homo-tetramer and comes from *Lactobacillus kefir* has a strong dependency on non-catalytic divalent magnesium ions for activity which is highly unusual for enzymes in this class[5 6] A role in allosteric regulation was ruled out since binding is slow and a role in maintaining structural integrity of the tetramer was also ruled out since the enzyme elutes as a tetramer even in the absence of Mg^{2+}. Two Mg^{2+} are bound per tetramer, both with an octahedral coordination shell of six oxygens (four from water molecules) with only one direct link to the enzyme being a monodentate coordinate bond to an oxygen on the C-terminal carboxylate of glutamine 251. The distance between the -OH of the catalytic tyrosine and the Mg^{2+} is considered too far (almost 17 Å) for Mg^{2+} to have a direct effect on catalysis. The main hypotheses as to its role are (i) an indirect effect on the conformation of the substrate binding loop via glutamine 251 indicating a role in substrate binding and product release and (ii) a role via a hydrogen bond network spanning from the Mg^{2+} coordination sphere ligands to the active site, which, if disturbed by removal of the Mg^{2+}, may disrupt the binding pocket and may also affect the unusual charge distribution found close to the active site.[6]

Section 3: Results of Preliminary Experiments Using W110A

The stereoinversion of R-alcohol to S-alcohol

The chronoamperogram corresponding to the initial stereoinversion experiment is shown in Figure S1 with the results of GC analysis presented in SI table 1. The electrode was loaded as described above with the loading solution containing 1.28 nmols of LK and 0.47 nmols of W110A. At the start of the experiment, the solution contains 4 mM R-alcohol and 5 mM Mg^{2+}; whilst holding at an oxidative potential of +0.21 V vs SHE, the coupled reaction was initiated by the addition of $NADP^{+}$ to a final concentration of 20 µM at t=0. A rapid increase in oxidative current resulted due to the combined activity of LK and W110A converting the R-alcohol to ketone. The current approached zero after 18 h and the charge passed during this time corresponded to a conversion of 89%. At this stage a sample was taken for chiral GC analysis and two interventions were made in real-time: the addition of EDTA to a final concentration of 25 mM (to chelate the Mg^{2+} ions to switch off the LK enzyme) and a potential switch to –0.64 vs SHE. A rapid increase in reductive current was observed as W110A now reduces the ketone produced during the initial oxidation stage. The reduction continues for approximately 18 h and the charge passed during this time corresponds to a conversion of 83.1% (from chiral GC analysis this was 82.3%).

The results of the chiral GC analysis indicate that the starting solution containing the R-alcohol also contains a small amount of ketone impurity that can be a result of oxidation of the alcohol in air. The conversion of the R-enantiomer during the oxidation phase, as analysed by chiral GC, was 91.7% (the discrepancy with the value obtained from the charge passed arising from baseline determination when calculating the area under the curve). In the reduction process, 87.7% of the ketone was converted to S-alcohol with a small amount of the R-alcohol produced. Overall, the cell solution which initially contained 3.93 mM R-alcohol was stereoinverted to contain 3.02 mM S-alcohol, with 0.52 mM R-alcohol and 0.45 mM ketone, still present; the enantiomer excess for S in the final bulk solution was 70.6% This initial result was promising considering the product inhibition of LK.

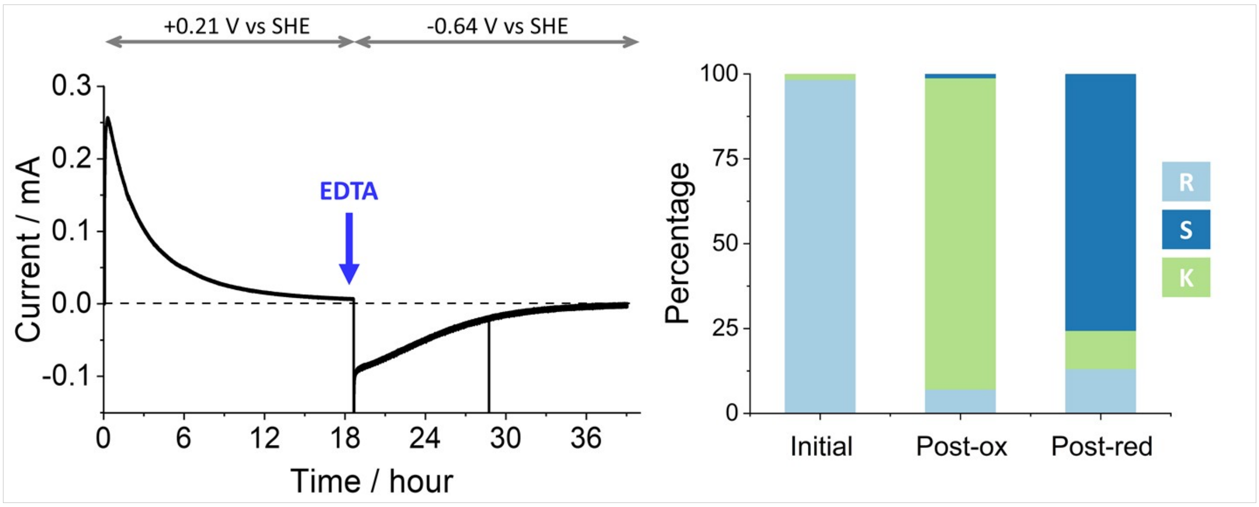

Figure S2. The stereoinversion of (R)-4-phenyl-2-butanol by W110A and LK nanoconfined in the e-Leaf monitored in real-time by chronoamperometry. Conditions: starting cell volume 5 mL and 1 mL sample taken at potential switch as indicated; solution stirred throughout using a magnetic flea; substrates: [(R)-4-phenyl-2-butanol] = 4 mM, [$NADP^+$] = 20 µM, [Mg^{2+}] = 5 mM; buffer: 50 mM TAPS pH 9.0; temperature: 25 ℃, Potential held at 0.21 V vs. SHE to drive oxidation during stage 1 and –0.64 V vs SHE for reduction. Experiments performed in an anaerobic glovebox to avoid any contribution to the current form the reduction of O_2. Enzyme loading: FNR loaded first by drop-casting, LK and W110A loaded by placing the FNR-loaded electrode in a stirred 2.5 mL solution containing 1.28 nmols of LK and 0.47 nmols of W110A in 50 mM TAPS buffer pH 9 at 4 °C for 18 h; electrode (Ti foil) SA: 7 cm^2.

Table S1. Chiral GC analysis corresponding to the experiment in Figure S2.

	[S]/mM	[R]/mM	[ketone]/mM	Solution composition during the time course			ee%
				ketone%	S%	R%	
**Start**	0.00	3.93	0.07	1.7	0.0	98.3	100.0 (R)
**Post-oxidation**	0.05	0.28	3.67	91.7	1.2	7.1	71.1 (R)
**Post-reduction**	3.02	0.52	0.45	11.3	75.5	13.1	70.4 (S)

The Deracemisation of Racemic Alcohol to S-alcohol

The chronoamperogram corresponding to the initial deracemisation experiment is shown in Figure S2. The starting solution contains 8 mM racemic alcohol and 5 mM Mg^{2+}. The electrode used in this experiment was loaded in the same way and with the same rations of enzymes, as used described for the experiment in Figure S1, with the loading solution containing 1.28 nmols of LK and 0.24 nmols of W110A. The first stage of the process is again an oxidation which was initiated by the addition of $NADP^+$ at t=0. After ~24 h, the current approached zero and from coulometry, the oxidation conversion at this stage was 68.9% (from chiral GC analysis this was 59.4%); the R-alcohol decreased from its initial concentration of 4 mM to 0.32 mM. The potential was then switched to a reductive one and EDTA was added in real-time to inactivate the LK enzyme. The reductive stage approached zero after a further 30 h and from coulometry, W110A, converted most of the ketone to S-alcohol with a conversion of 88.6%. The discrepancy between the expected conversion based on the coulometry (from the oxidation phase, 68.9%) and from GC analysis (post-oxidation phase, 59.4%) can be explained by the cross-over of product to the side-arm of the electrochemical cell, despite separation by a glass frit. A sample from the side arm was analysed at the end of the experiment post-reduction phase and the results are listed in table 3.

The final ee is 85.8%, proof of concept that the e-Leaf used in this way achieves controlled deracemisation.

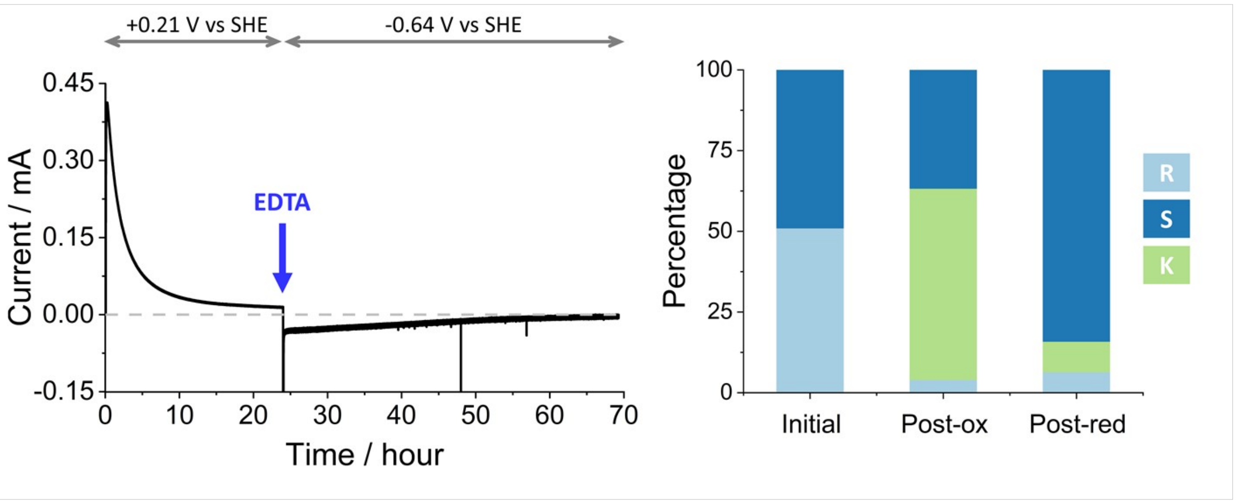

Figure S3: The deracemisation of (±)-4-phenyl-2-butanol solution by W110A and LK nanoconfined in the e-Leaf and monitored in real-time by chronoamperometry. **Conditions:** starting cell volume 5 mL and 1 mL sample is taken at potential switch as indicated; solution stirred throughout using a magnetic flea; substrate [(±)-4-phenyl-2-butanol] = 8 mM, $[NADP^+]$ = 20 µM, $[Mg^{2+}]$ = 5 mM buffer: 50 mM TAPS pH9.0; temperature: 25 ℃, Potential held at +0.21 V vs. SHE to drive oxidation and –0.64 V for reduction. Experiments performed in an anaerobic glovebox to avoid any contribution to the current from the reduction of O_2. Enzyme loading: FNR loaded first by drop-casting, LK and W110A loaded after FNR, by stirring in a 2.5 mL solution containing 1.28 nmols of LK and 0.24 nmols of W110A in 50 mM TAPS buffer pH 9 at 4 °C for 18 h; electrode (Ti foil) SA: 14 cm^2.

Table S2. Chiral GC analysis corresponding to the experiment in Figure S3.

	[S]/mM	[R]/mM	[ketone]/mM	Solution composition during the time course			ee%
				ketone%	S%	R%	
**Start**	3.92	4.08	0.00	0	49.0	51.0	2 (R)
**Post-oxidation**	2.94	0.32	4.74	59.3	36.7	4.0	80.3 (S)
**Post-reduction**	4.82	0.37	0.54	9.5	84.1	6.4	85.7 (S)
**Side-arm**	2.27	1.44	0.14	30.2	63.5	6.3	81.9(S)

It is worth noting the low current achieved during the reduction phase: when starting with a racemic mixture, the aim is to turn most of the R enantiomer into ketone during the initial oxidation stage, and therefore the LK enzyme must be working maximally compared to the W110A enzyme. When the LK is switched off and the reduction phase begins, the W110A is now required to work maximally to convert the ketone produced during the first stage, to S-alcohol; this means that its loading cannot be too low (which would satisfy the first oxidation stage). Both enzymes suffer from product inhibition which adds another layer of complexity.

These experiments indicated that the conversion of ketone during the reduction phase, was incomplete and unusually, that the amount of R-alcohol increased slightly thus affecting the ee. This latter phenomenon was noted in our previous two-electrode approach[2] and was explained by the nanoconfinement of enzymes and reactants which accelerates their interactions thus favouring equilibrium and therefore racemisation. In this work, additional factors which also would result in the production of R-alcohol include incomplete chelation of magnesium by EDTA and therefore incomplete deactivation of the LK enzyme or, an inadequate selectivity by W110A for the S enantiomer. We therefore took steps to address these issues. Firstly, both the oxidation and reduction phases should run to completion; oxidation is favoured at a high pH whilst reduction is favoured at lower pH values. We attribute the incomplete consumption of ketone to the sub-optimal pH which had been set for the first oxidation stage at pH 9. To address this, a simple adjustment to the bulk solution before starting phase two was carried out, made possible by the properties of the e-Leaf.

Secondly, the W110A variant was replaced with W110V (despite its slower rate) which has superior selectivity for the S-enantiomer. The slower rate is easily compensated for by loading more of this variant in the electrode pores, moreover, the fine tuning of the relative amounts of all three enzymes achieves optimal activity. To ensure the complete inactivation of the LK enzyme, the addition of EDTA, which previously took place in real-time, was allowed to incubate without an applied potential for 1-hour between the oxidation and reduction phases to allow for the complete chelation of all Mg^{2+} ions, thus switching off LK completely before driving the reduction.

Section 4: Chiral GC Analysis for Figure 2 and Figure 3 in the Main Manuscript

Enantiomer analysis for the stereoinversion experiment shown in Figure 2

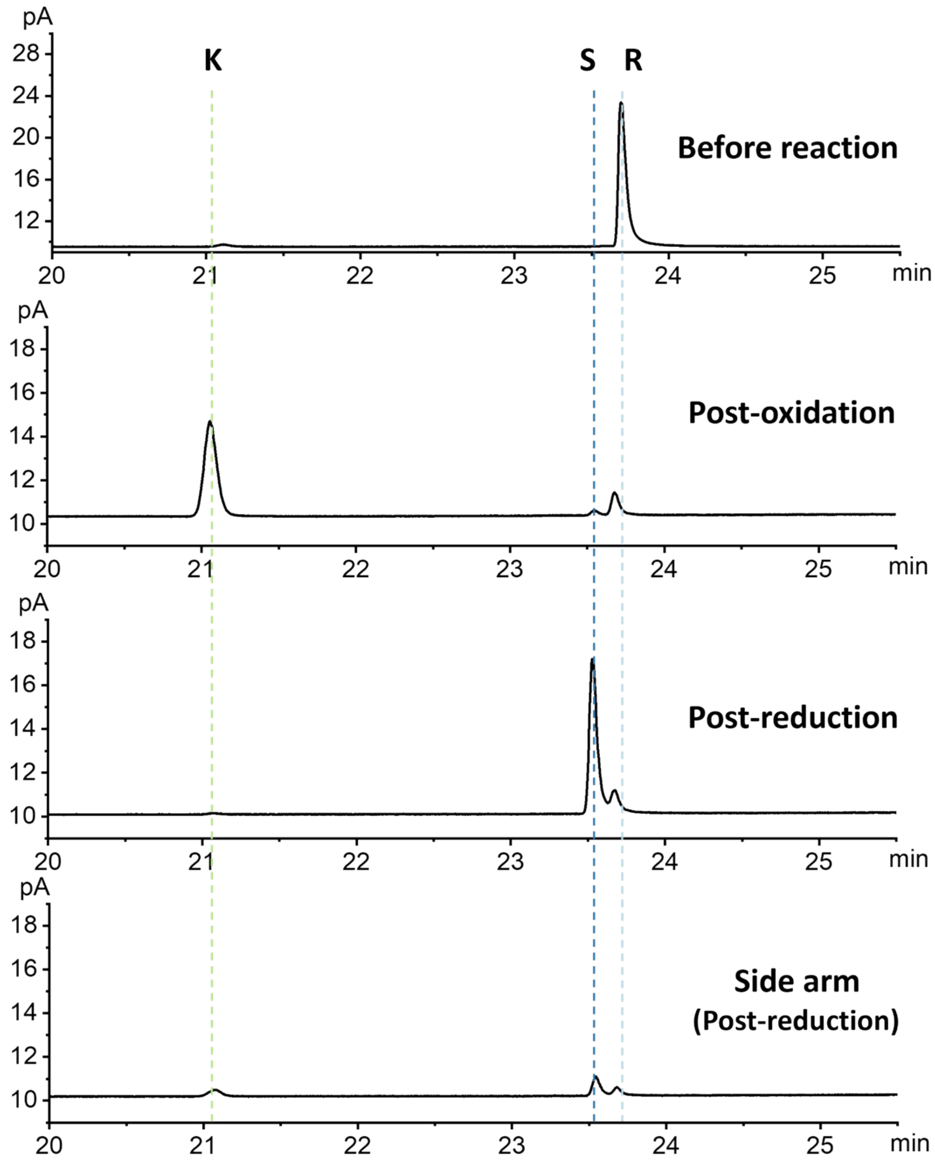

Figure S4. GC-FID analysis signal for the stereoinversion experiment shown in Figure 1

Table S3: GC Enantiomer analysis of the bulk solution (post-oxidation and post-reduction) and of the solution in the auxiliary side-arm (post-reduction) for the stereoinversion experiment shown in Figure 2

		[S]/mM	[R]/mM	[ketone]/mM	Solution composition during the time course			ee%
					ketone %	S%	R%	
**Bulk Solution**	Start	0.00	3.93	0.07	1.7	0.0	98.3	100 (R)
	Post-oxidation	0.09	0.48	3.43	85.7	2.3	12.0	66.7 (R)
	Post-reduction	2.78	0.50	0.04	1.1	83.9	15.0	69.5 (S)
**Side-arm**	Post-reduction	0.34	0.16	0.19	27.7	49.2	23.1	36 (S)

Enantiomer analysis for the deracemisation experiment shown in Figure 3

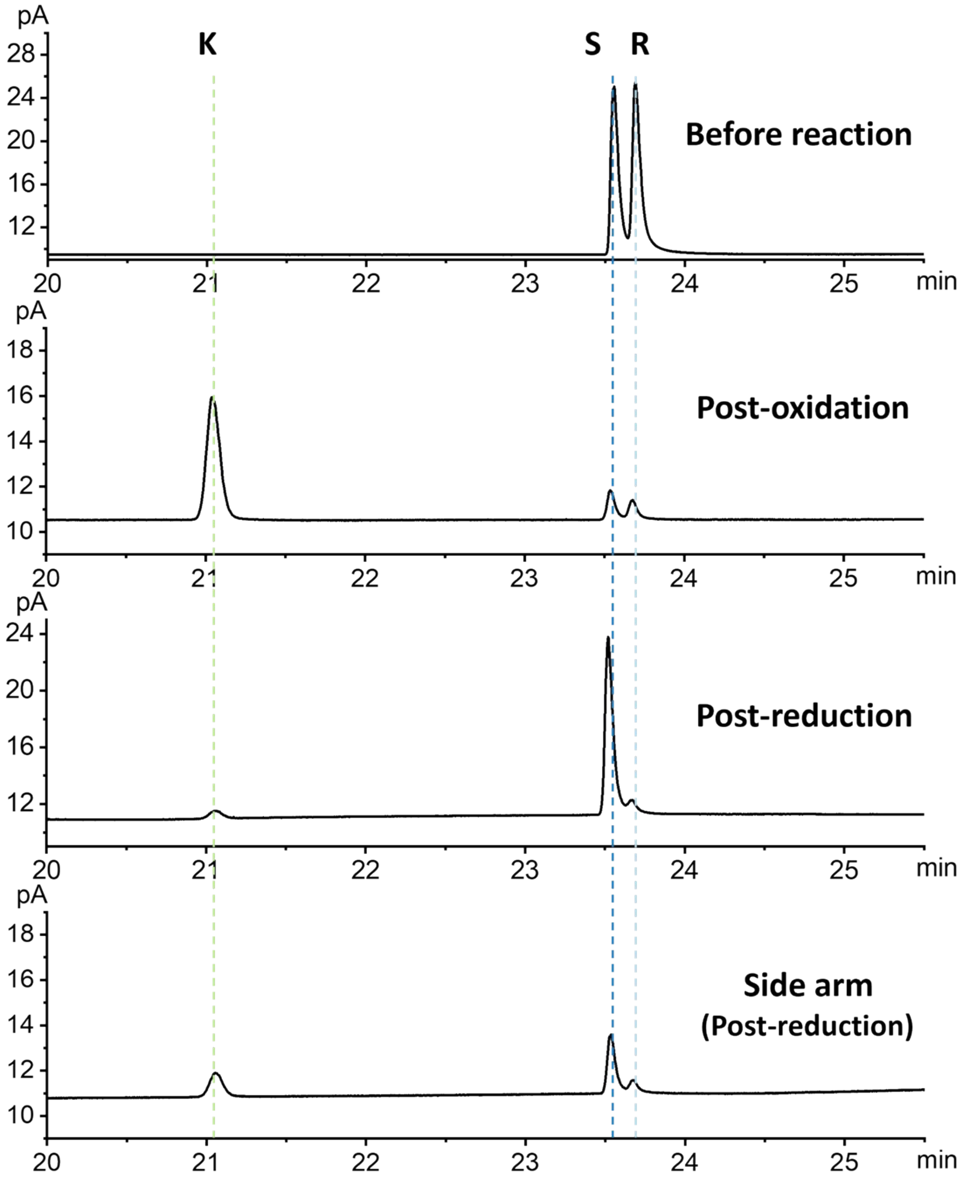

Figure S5. GC-FID analysis signal for the stereoinversion experiment shown in Figure 3

Table S4. GC Enantiomer analysis of the bulk solution (post-oxidation and post-reduction) and of the solution in the auxiliary side-arm (post-reduction) for the deracemisation experiment shown in Figure 3

		[S]/mM	[R]/mM	[ketone]/mM	Solution composition during the time course			ee%
					ketone %	S%	R%	
**Bulk Solution**	Start	3.92	4.08	0.00	0.0	49.0	51.0	2 (R)
	Post-oxidation	0.95	0.73	6.31	78.9	11.9	9.2	12.9 (S)
	Post-reduction	5.06	0.50	0.33	5.6	86.0	8.5	82.1 (S)
**Side-arm**	Post-reduction	1.10	0.31	0.71	33.7	51.9	14.4	56.5 (S)

Section 5: Control experiments to establish that the Zinc-Dependent variants, W110A and W110V, remain active in the presence of 25 mM EDTA.

Control chronoamperometry experiments in which the zinc-dependent W110A and W110V variant ADH enzymes from *T. ethanolicus* and the Magnesium-dependent *L. kefir* enzyme (LK) were each subjected to 25 mM EDTA, are shown in Figure S6 (A-C). An electrode pre-loaded with FNR (by drop-casting followed by rinsing) was placed in a starting solution containing 8 mM racemic alcohol; $NADP^+$ (20 µM for experiments A and B and 10 µM for experiment C) was also added before the reaction was initiated by the real-time addition of each ADH to the bulk solution, as indicated on each graph. The current increases as enzyme molecules adsorb into the electrode pores where they couple to the rapid bidirectional interconversion of $NADP^+$/NADPH by FNR, to catalyse oxidation of the alcohol. As the current approached a maximum, the bulk solution (still containing ADH enzyme) was replaced in real-time with fresh solution containing the same concentrations of alcohol and $NADP^+$, but without enzyme, thus the current observed after this buffer exchange no longer increased (note that the considerable physical disruption during buffer exchange has likely caused some erosion of the electrode surface which has caused a decrease in current for experiment shown in C). In real-time, EDTA was then added to the bulk solution to a final concentration of 25 mM as indicated on each graph. In the experiments using the zinc-dependent variants (Figure S6 A and B), the current was unaffected by this addition, indicating that each variant remained active, and, since their activity is dependent on active site zinc, we can conclude that EDTA at a concentration of 25 mM was unable to remove it. This resistance to EDTA is not unexpected, as seminal work on the binding of catalytic zinc to ADH enzymes has shown.[7] . In contrast, the addition of EDTA (25 mM) completely deactivates the magnesium-dependent LK enzyme (Figure S6 C).

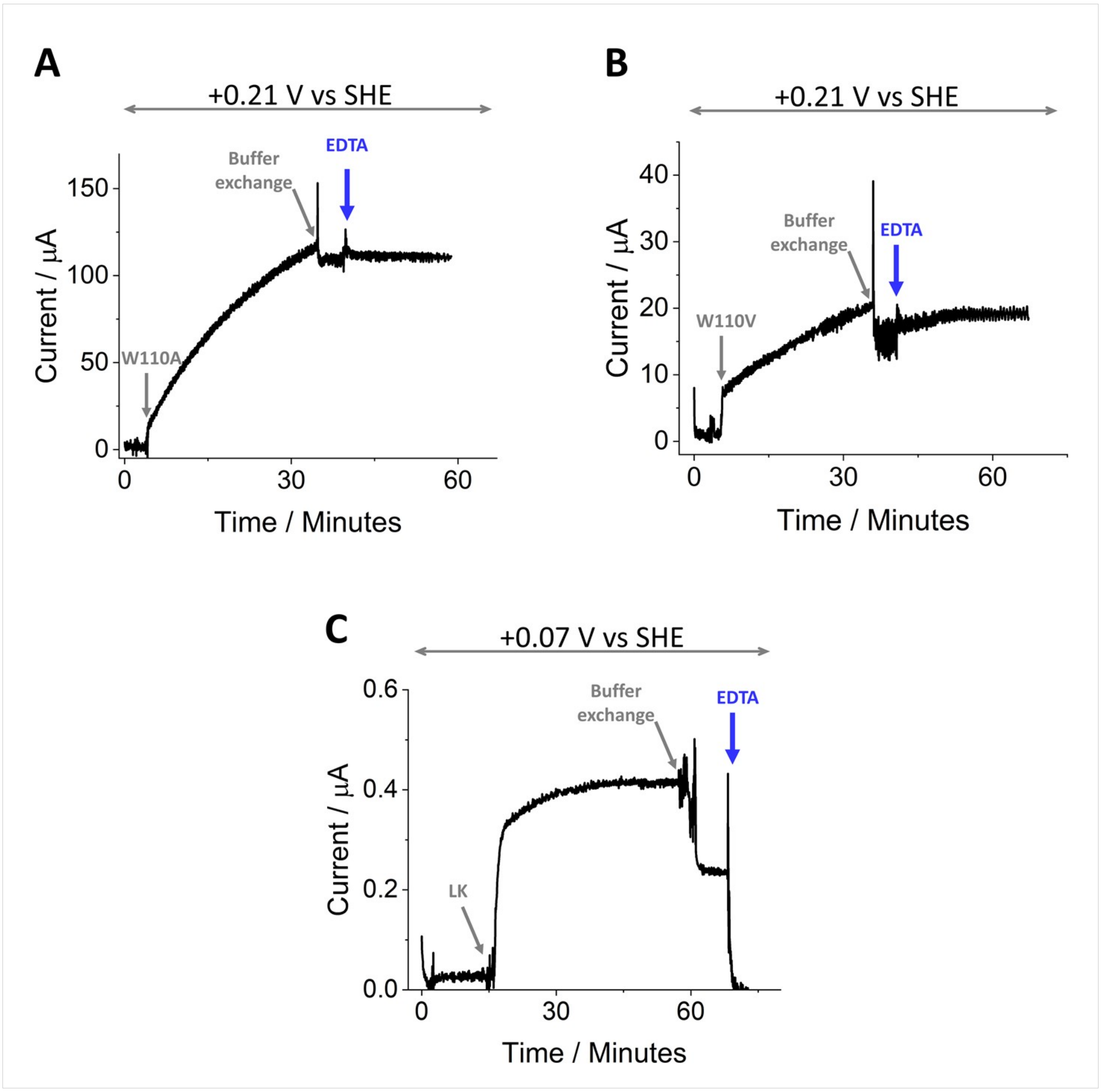

Figure S6: The effect of 25 mM EDTA on the activity of ADH variants, W110A and W110V, from *T. ethanolicous* and LK from *L. Kefir*. The oxidation of (±)-4-phenyl-2-butanol catalysed by **A:** W110A, **B:** W110V, and **C:** LK with real-time addition of EDTA to a final concentration of 25 mM as indicated. For the experiments in **A** and **B**, an ITO@Ti foil electrode (3.5 cm^2) was preloaded with FNR by drop-casting followed by rinsing to remove excess enzyme; for the experiment in **C**, FNR was preloaded in the same way, but the electrode used was an ITO@graphite (0.07 cm^2). The starting solution for all three experiments contained 8 mM racemic alcohol; $NADP^+$ (20 µM for **A** and **B** and 10 µM for **C**) was also introduced before the addition of each variant ADH to the bulk solution as shown. A buffer exchange to remove any ADH enzyme remaining in the bulk solution, was carried out as indicated, replacing the solution with fresh buffer containing only racemic alcohol and $NADP^+$ at the same initial concentrations. **Conditions** Reaction volume: 4 mL; for **A** and **B** the solution was stirred throughout using a magnetic flea, for **C**, the electrode was rotated at 1000 rpm; Magnesium (5 mM) was included in experiment C only; buffer: 50 mM TAPS pH 9.0; temperature: 25 ℃; **A** and **B**: potential held at +0.21 V vs. SHE, **C**: potential held at +0.07 V vs SHE. Experiments performed in an anaerobic glovebox to avoid any contribution to the current from the reduction of O_2.

References

1. A. Bard, J. and Faulkner, L., R., *Electrochemical Methods : Fundamentals and Applications*, Wiley, Chichester, 2nd edn., 1980.
2. L. Wan, R. S. Heath, C. F. Megarity, A. J. Sills, R. A. Herold, N. J. Turner and F. A. Armstrong, *ACS Catalysis*, 2021, **11**, 6526-6533.
3. R. S. Heath, J. J. Sangster and N. J. Turner, *ChemBioChem*, 2022, **23**, e202200075.
4. C. F. Megarity, B. Siritanaratkul, B. Cheng, G. Morello, L. Wan, A. J. Sills, R. S. Heath, N. J. Turner and F. A. Armstrong, *ChemCatChem*, 2019, **11**, 5662-5670.
5. W. Hummel, *Applied Microbiology and Biotechnology*, 1990, **34**, 15-19.
6. K. Niefind, J. Müller, B. Riebel, W. Hummel and D. Schomburg, *Journal of Molecular Biology*, 2003, **327**, 317-328.
7. J. H. R. Kägi and B. L. Vallee, *Journal of Biological Chemistry*, 1960, **235**, 3188-3192.
